# Characterization by Small RNA Sequencing of Taro Bacilliform CH Virus (TaBCHV), a Novel Badnavirus

**DOI:** 10.1371/journal.pone.0134147

**Published:** 2015-07-24

**Authors:** Syeda Amber Kazmi, Zuokun Yang, Ni Hong, Guoping Wang, Yanfen Wang

**Affiliations:** 1 State Key Laboratory of Agromicrobiology, Huazhong Agricultural University, Wuhan, Hubei, China; 2 College of Plant Science and Technology, Huazhong Agricultural University, Wuhan, Hubei, China; University of Pittsburgh School of Medicine, UNITED STATES

## Abstract

RNA silencing is an antiviral immunity that regulates gene expression through the production of small RNAs (sRNAs). In this study, deep sequencing of small RNAs was used to identify viruses infecting two taro plants. Blast searching identified five and nine contigs assembled from small RNAs of samples T1 and T2 matched onto the genome sequences of badnaviruses in the family *Caulimoviridae*. Complete genome sequences of two isolates of the badnavirus determined by sequence specific amplification comprised of 7,641 nucleotides and shared overall nucleotide similarities of 44.1%‒55.8% with other badnaviruses. Six open reading frames (ORFs) were identified on the plus strand, showed amino acid similarities ranging from 59.8% (ORF3) to 10.2% (ORF6) to the corresponding proteins encoded by other badnaviruses. Phylogenetic analysis also supports that the virus is a new member in the genus *Badnavirus*. The virus is tentatively named as *Taro bacilliform CH virus* (TaBCHV), and it is the second badnavirus infecting taro plants, following *Taro bacilliform virus* (TaBV). In addition, analyzes of viral derived small RNAs (vsRNAs) from TaBCHV showed that almost equivalent number of vsRNAs were generated from both strands and the most abundant vsRNAs were 21 nt, with uracil bias at 5' terminal. Furthermore, TaBCHV vsRNAs were asymmetrically distributed on its entire circular genome at both orientations with the hotspots mainly generated in the ORF5 region.

## Introduction

Taro (*Colocasia esculenta* L. Schot) is an ancient crop cultivated for its edible corms, and leaves. Due to its vegetative propagation through tubers, viruses are easy to be transmitted to next generations and dispersed worldwide by planting and transferring viral infected tubers. To date, six viruses infecting taro plants have been reported [1–6]. A taro badnavirus, name as *Taro bacilliform virus* (TaBV), was firstly reported in Papua New Guinea (PNG) [3]. The presence of a badnavirus in taro plants grown in China was confirmed by polymerase chain reaction (PCR) using degenerate primers [7].

Viruses in the genus *Badnavirus* have been gaining attention globally and are currently considered as an economically important plant pathogen since they can cause destructive losses to many crops [8, 9]. Badnaviruses have striking features, including the capacity to integrate into host genomes [10], infection on a wide range of tropical, sub-tropical, and temperate crops [11] and high variability at both genomic and serological levels [12]. Badnaviruses are characterized by non-enveloped bacilliform particles (120‒150 × 30 nm), which contain a circular, double-stranded DNA (dsDNA) genome of 7−8 kb in size [13]. The typical genomes of badnaviruses contain three open reading frames (ORFs) on the plus strand [14]. ORF1 encodes a small and function unknown protein, ORF2 encodes a virion-associated protein. ORF3 encodes a large polyprotein, which is cleaved into the movement protein (MP), coat protein (CP), aspartic protease (AP), reverse transcriptase (RT) and ribonuclease H (RNase H) [15–18]. Moreover, some badnaviruses have more ORFs, including four ORFs for TaBV [3], *Piper yellow mottle virus* (PYMoV) [19], *Sweetpotato badnavirus* (SPBV-A) and *Sweetpotato badnavirus B* (SPBV-B) [20], five for *Cacao swollen shoot virus* (CSSV) [21], *Pagoda yellow mottle associated virus* (PYMAV) [22], *Rubus yellow net virus* (RYNV) [23], six for *Citrus yellow mosaic virus* (CYMV) [9], and seven for *Draceana mottle virus* (DrMV) [24]. However, these additional ORFs are within or largely overlapped with ORF3 [9], except for ORF7 of DrMV.

RNA silencing is an antiviral immunity and fundamental cellular mechanism that regulates gene expression through the production of small RNAs (sRNAs) [25]. High-throughput sequencing of small RNA combined with bioinformatics analysis has shown great potential for the identification and genome reconstruction of known and unknown plant viruses and viroids [26–28], as well as insect viruses [29, 30]. In the present study, we used deep sequencing of sRNAs combined with viral sequence specific amplification to construct the complete genome of a novel badnavirus infecting taro plants. The virus derived small RNA (vsRNA) profile was evaluated.

## Materials and Methods

### Plant Materials

Leaf samples of two taro plants (T1 and T2) were used for sequencing of sRNAs. Taro plants were collected from two taro fields in Hubei Province in central China. All sample collections were done with approval from local institutes, and no specific permissions were required for these locations/activities. The study did not involve endangered or protected species. Those plants maintained in pots in an insect proof glasshouse for continuous supervision of viral diseases. Previous RT-PCR tests indicated that the two plants were positive for a badnavirus [7]. At the greenhouse, a mild feathery mosaic symptom on young leaves and brown spots on matured leaves were observed.

### Total RNA extraction and deep sequencing of sRNAs

For deep sequencing of sRNAs, young leaves were collected during the growing season. Total RNA was extracted from two leaf samples using Trizol reagent (Invitrogen, Carlsbad, CA, USA). The sRNA libraries were constructed at Biomarker Technologies Company Beijing, China by using the ‘NEB multiplex Small RNA Library’ kit (New England BioLabs), following the manufacturer’s recommendations. Briefly, sRNA molecules (<30 nt) were isolated by polyacrylamide gel electrophoresis (PAGE), the 3' end was ligated with an adaptor, and with the addition of a RT primer, 5' ligation was conducted. Adaptors ligated to the sRNAs were converted into cDNA, amplified by PCR, and recovered by using 6% PAGE, then sequenced an Illumina HiSeq™ 2000 platform (Illumina, Inc., San Diego, CA, USA).

### Small RNAs sequence assembly

The resulting raw reads from deep sequencing were processed to trim the adaptor sequences, followed by assembling into contigs using Velvet software 0.7.3 [31] with a k-mer value of 17. The contigs were scanned against the GenBank database (http://www.ncbi.nlm.nih.gov/) using BLASTN and BLASTX to search for similar sequences.

### Primer designing

Initially, eight sets of primers (S1 Table) were designed based on the assembled contig sequences using Oligo7 [32]. The amplified fragments using those primer pairs covered almost the whole genome of the virus, with a few gaps. To ensure that the obtained sequences were derived from the same viral genome, seven sets of primers that were designed based on the sequences amplified using the eight primer pairs were used to amplify the full genome of the virus. All seven fragments overlapped each other by at least 50 nucleotides (nts).

### Total DNA extraction and amplification of complete viral genome

Total DNA was extracted from leaf tissues of taro samples T1 and T2, respectively, by using the hexadecyltrimethylammonium bromide (CTAB) method [33] and digested with 1 μL RNase A (10 mg/mL). PCR reactions were conducted in a 50 μL-reaction volume consisting of 10 × buffer with 15mM MgCl_2_, 25 mM MgCl_2_, 10 mM dNTPs, 1U Taq polymerase, 100 M each of forward and reverse primers, 50 ng DNA, and sterile Milli-Q water to a final volume. The PCR products were separated in a 1% agarose gel, isolated with the AxyPrep™ DNA gel extraction kit (Axygen Bioscience, Hangzhou, China), and inserted into a pMD-18-T vector (Takara, China). At least five clones of each product were sequenced at Sangon Biological Engineering & Technology and Service Co. Ltd, Shanghai, China).

### Genome assembly and sequence analysis

The obtained sequences were assembled into a contiguous sequence at a standard of ≥ 99.9% similarity at each overlapping region using DNAMAN Version 6.0 (Lynnon Biosoft, Montreal, QC, Canada). ORF finder (http://www.ncbi.nlm.nih.gov/projects/gorf/) was used to identify putative ORFs in the viral genome. Deduced amino acid (aa) sequences were analyzed for conserved protein domains (CDD) (http://www.ncbi.nlm.nih.gov/structure/cdd.shtml) and theoretical molecular weights were calculated by using ExPASy (http://web.expasy.org/compute_pi/).

### Phylogenetic analysis

The sequences of 20 badnaviruses and one tungrovirus of family *Caulimoviridae* were retrieved from NCBI (http://www.ncbi.nlm.nih.gov/). Phylogenetic analyses were performed using the neighbor-joining method in MEGA 6.0 [34] and were rooted to the corresponding sequence of *Rice tungro bacilliform virus* (RTBV). The virus names and sequence accession numbers used for the analysis are listed in S2 Table. Robustness of nodes of the phylogenetic tree was assessed from 1,000 bootstrap resampling, and values ≥ 70% were used as labels for internal nodes of both trees.

### vsRNA profile

Reads of vsRNA were mapped to the virus genome using the Bowtie (1.0) software [35] (http://sourceforge.net/projects/bowtie-bio/files/) and only those having sequences identical or complementary to the viral genomic sequence with < 2 mismatches were identified as vsRNAs.

## Results

### Small RNA characterization and the amplification of the full genome of a novel badnavirus

**Fig 1:**
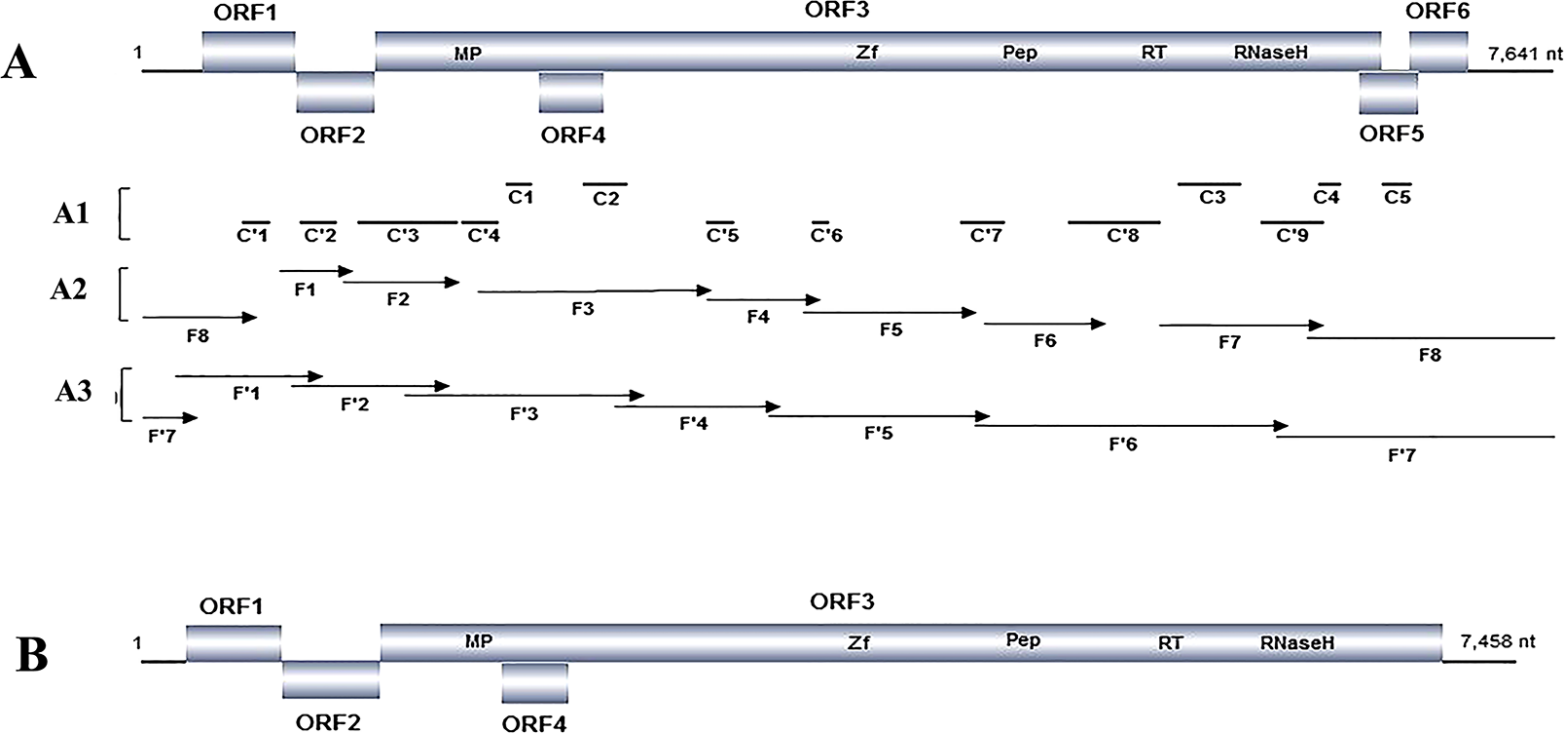
Genome organization of *Taro bacilliform* CH *virus* (TaBCHV). The putative ORFs of TaBCHV are indicated by rectangles, domains identified within ORF 3 are shown (A), contigs obtained from samples T1 (C1– C5) and T2 (C'1–C'9) are presented by black lines (A1), and fragments F1–F8 amplified from the first cycle of PCR (A2) and F'1–F'7 amplified from the second cycle of PCR (A3) are represented by arrows. The genome organization of *Taro bacilliform virus* (TaBV) (B) is outlined to show its difference with that of TaBCHV.

A total of 15,748,273 and 11,425,217 raw reads were obtained from samples T1 and T2, respectively. After removing adapter sequences and selecting by size differences, 9,348,325 and 9,959,864 clean reads with sizes within the range of 18−26 nts were generated from the two samples. These sRNAs were assembled by using the Velvet software. The resulting contigs were searched using BLASTN and BLASTX [36] against NCBI GenBank. BLAST results showed that five (C1–C5) and nine (C'1–C'9) contigs from samples T1 and T2 matched to the genome sequences of badnaviruses of family *Caulimoviridae*, respectively (Fig 1A1), with the highest amino acid (aa) similarity of 70% to the corresponding regions of CYMV (NC_003382). In addition, seven contigs from T1 and fifteen contigs from T2 matched to the *Dasheen mosaic virus* (DsMV) in the family *Potyviridae*. Here, only a badnavirus was considered.

Eight sets of primers were designed based on the obtained badnavirus contigs, which were homologous to CYMV sequences. The target fragments (F1–F8) amplified from both samples by using the designed primers were separately sequenced and assembled into large fragments (Fig 1A2). To fill the gaps between the fragments produced by using those primers and to avoid mismatches across each overlapping region, other seven sets of primers were designed based on the obtained sequences. All amplified fragments (F'1–F'7) in the second cycle of the PCR reactions crossed the overlapping regions of fragments obtained in the first cycle of the PCR reactions (Fig 1A3). Sequencing results showed that fragments obtained by two cycles of PCR reactions showed >99% sequence similarity in the corresponding regions. Finally, the full genome sequences of the badnavirus from two samples were assembled. Here, we tentatively named the novel virus as Taro bacilliform CH virus (TaBCHV).

### Genome characterization and sequence analysis of the two isolates of TaBCHV

The genome sizes of TaBCHV isolates, TaBCHV-1 and TaBCHV-2 (GenBank Accession Nos: KP710178 and KP710177) were 7,641 bp, which was within the range of badnavirus genomes. These two isolates shared 98% overall genomic nucleotide identity. Pairwise comparison of genome sequences of TaBCHV-1 and TaBCHV-2 with other reported badnaviruses showed genomic similarities ranging from 44.1% with RYNV to 55.8% with *Fig badnavirus* (FBV). Six ORFs (Fig 1A) were identified on the plus strand. Then, the genome structure of the virus differed from that of TaBV, which has four ORFs (Fig 1B). The ORF1, ORF2, ORF3, ORF4, ORF5, and ORF6 between two isolates shared 97.6%, 93.9%, 97.3%, 100, 100%, and 100% nucleotide identities, respectively.

All six ORFs of TaBCHV start with an ATG codon and terminate either with a TGA stop codon (ORF1, ORF2, ORF3, ORF5, and ORF6) or a TAA codon (ORF4), and five of these overlapped with each other, except for ORF4, which lies within ORF3.

### Non-coding regions of TaBCHV genomic DNA

The intergenic region (IR) of TaBCHV comprised 981 nts and has conserved nucleic acids, as earlier described for dsDNA viruses [37]. Within the IR, a putative tRNA^met^ binding region was detected at position 1‒18 nt (5'-TGGTATCAGAGCTTTGTT-3') with 16 out of the 18 nts complementary to the consensus sequences of plant tRNA^met^ (3'-ACCAUAGUCUCGGUCCAA-5') which has been previously described as one of the priming sites for reverse transcription [17]. There is a potential TATA box (TATAAA) located at position 7,503‒7,508 nt, which was identical to that of the CSSV, and a downstream poly adenylation signal (AAAATAA) at position 7,624‒7,630 nt. However, the polyadenylation signal was not detected in the TaBV genome [3].

### Coding regions

ORF1 (384–821 nt) of TaBCHV potentially encodes for a 145 aa protein with a predicted molecular weight (MW) of 16.8 kDa (S3 Table). The predicted protein has 15.2%‒56.8% similarity with the corresponding proteins of other badnaviruses (Table 1). ORF1 contains a domain of unknown function (DUF), named as DUF1319 in Pfam database, which is restricted to badnaviruses [38, 39].

**Table 1:** Comparison of the nucleotide of genomes and amino acid sequences of six ORFs of TaBCHV-2 with the corresponding sequences of TaBCHV-1 and other badnaviruses.

Viruses	Genome size (bp)	Nucleotide similarity (%)	Amino acid similarity (%)					
			ORF1	ORF2	ORF3	ORF4	ORF5 ^a^	ORF6 ^b^
TaBCHV-1	7,641	98.2	97.6	93.9	97.3	100	100	100
TaBV	7,458	45.5	35.2	23.7	44.6	9.1	ND	ND
CSSV	7,161	52.4	53.6	33.3	56.4	6.1	25.5	ND
CYMV	7,559	53.5	56.8	46.5	58.4	20.2	10.8	ND
DsBV	7,261	52.5	40.8	39.5	54.7	ND	ND	ND
DrMV	7,531	47.0	42.4	31.6	47.6	10.1	16.7	14.3
FBV	7,140	55.8	56.0	43.0	59.8	ND	ND	ND
HBV	7,435	54.0	52.8	46.5	56.7	9.1	ND	ND
PYMoV	7,562	54.7	45.6	47.4	59.5	ND	11.8	ND
PYMAV	7,424	45.7	15.2	21.9	43.7	ND	8.8	ND
RYNV	7,932	44.1	19.2	28.9	43.6	ND	14.7	10.2

^**a**^ Based on the position of the ORF along the genome, the ORF5 of TaBCHV-2 was compared to CSSV ORFY, CYMV ORF6, DrMV ORF6, PYMoV ORF4, PYMAV ORF4 and RYNV ORF4.

^**b**^ The ORF6 of TaBChV-2 was compared to DrMV ORF7 and RYNV ORF 5.

ND, No Corresponding ORF.

ORF2 (818–1,198 nt) encodes for a 126 aa protein with a MW 14.1 kDa. It shares 21.9%‒47.4% aa similarity with the corresponding proteins encoded by other badnaviruses.

**Fig 2:**
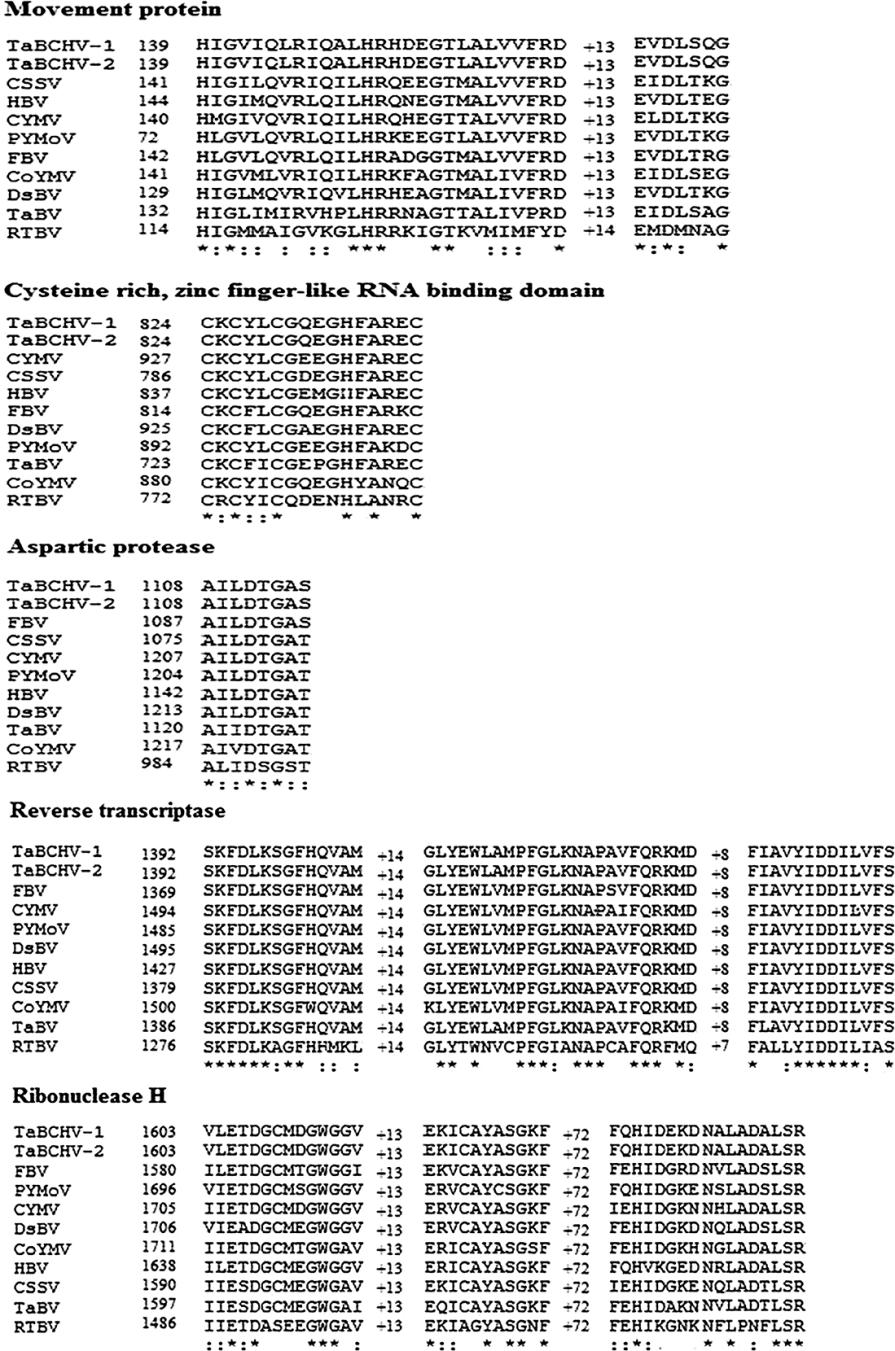
Comparison of amino acid sequences of domains highly conserved in the polyproteins encoded by ORF3 of badnaviruses and a tungrovirus. The virus names and the positions of starting amino acid are indicated before each sequence. Identical (*) and conserved (:) amino acids are marked.

ORF3 (1,198–6,609 nt) encodes for a 1,803 aa polyprotein with a MW of 206.4 kDa, which is slightly smaller than the corresponding protein of known badnaviruses. The protein showed highest similarity of 59.8% with the polyprotein encoded by FBV (Table 1). It harbors domains homologue to those of MP, AP, RT and RNase H, and a zinc finger like RNA binding domain (CXCX2CX4HX4C), which are highly conserved in the polyproteins of badnaviruses (Fig 2).

ORF4 (2,096–2,455 nt) is located within ORF3, encodes for a 119 aa hypothetical protein with a MW 13.2 kDa, and shares highest similarity of 20.2% with that of CYMV. Counterparts of TaBCHV ORF4 have been detected in CSSV, CYMV, DrMV, HBV, TaBV, PYMoV, and RYNV and SVBV-A and SVBV-B.

ORF5 (6,530–6,838 nt) partially overlaps with the C-terminal region of ORF3. The position of ORF5 is similar to that of ORF4 of PYMoV, PYMAV and RYNV, ORF Y of CSSV, and ORF6 of CYMV and DrMV. It encodes for a 102 aa protein with an MW 11.9 kDa, and shares highest similarity (25.0%) with CSSV.

ORF6 (6,720–7,043 nt), which is located downstream of ORF5, potentially encodes for a 107aa protein, with a MW 12.4 kDa. Previously, ORF7 of DrMV was identified at similar position. ORF6 of TaBCHV and ORF7 of DrMV showed 14.3% aa similarity.

### Phylogenetic analysis

**Fig 3:**
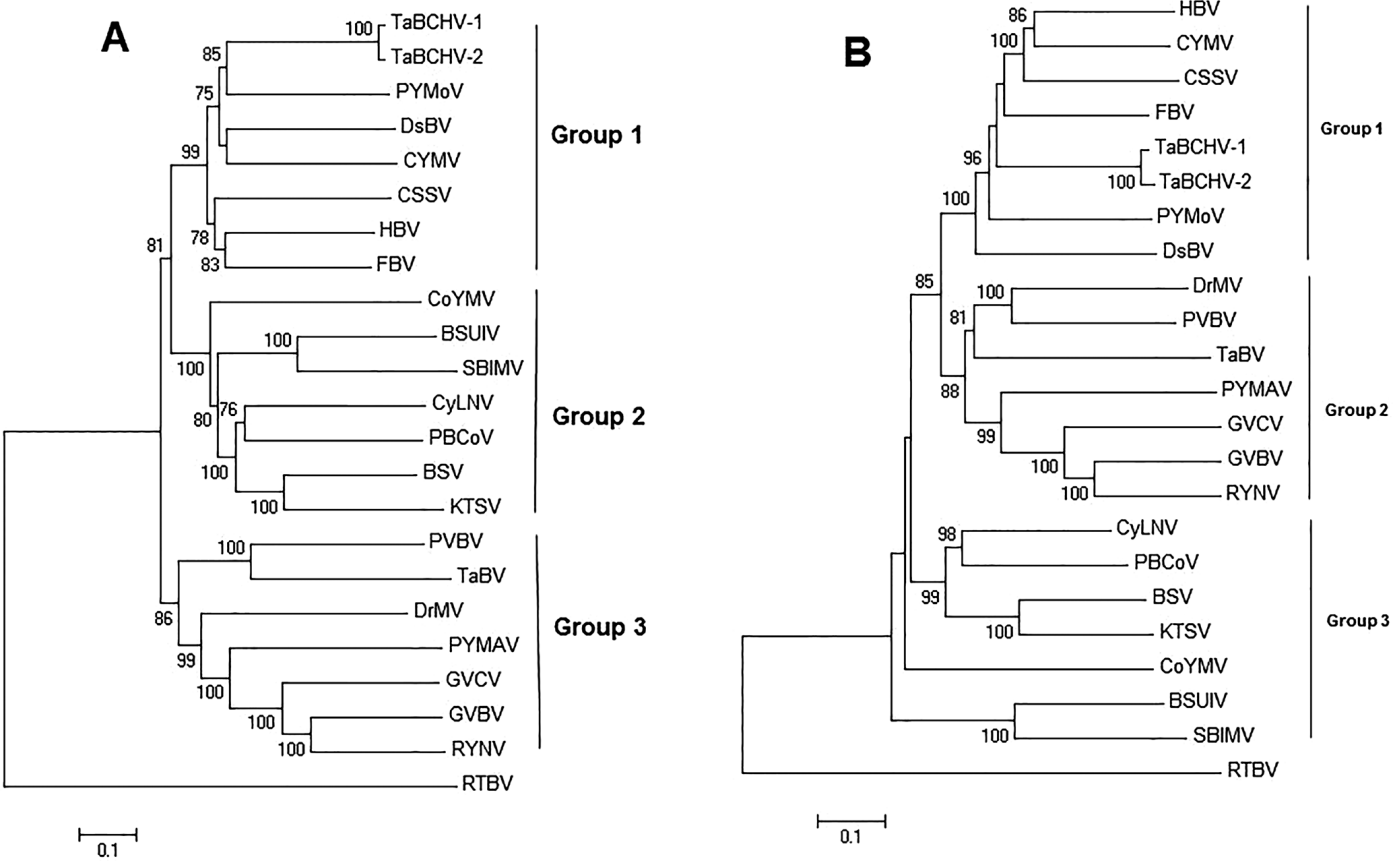
Neighbor-joining phylogenetic trees of badnaviruses generated from the full genomic sequences (A) and putative amino acid sequences of ORF3 (B). The phylogenetic trees were rooted by using the genome sequence of *Rice tungro bacilliform virus* (RTBV) (A) and the polypeptide of RTBV (B). Branch lengths are proportional to genetic distances. Numbers at the nodes of the branches represent bootstrap values (1000 replicates).

Phylogenetic relationships between the two TaBCHV isolates and other badnaviruses were estimated basing on their full genome sequences (Fig 3A) and aa sequences of ORF3 (Fig 3B). The two phylogenetic trees had similar topology structures, and all tested viruses were clustered into three major groups, namely groups 1‒3. In both phylogenetic trees, TaBCHV isolates consistently had the same phylogenetic positions with CSSV, CYMV, DsBV, FBV, HBV, and PYMoV in the group 1, but was distant from TaBV.

The results, together with sequence and structure comparisons of the full genomes, indicate that the virus evaluated in the present study is a new member of the genus *Badnavirus* and the second badnavirus that has been determined to infect taro plants. The two badnaviruses, TaBCHV and TaBV, show differences in genome structure and RT/RNase sequences, with < 80% similarity.

### vsRNA profiling

**Fig 4:**
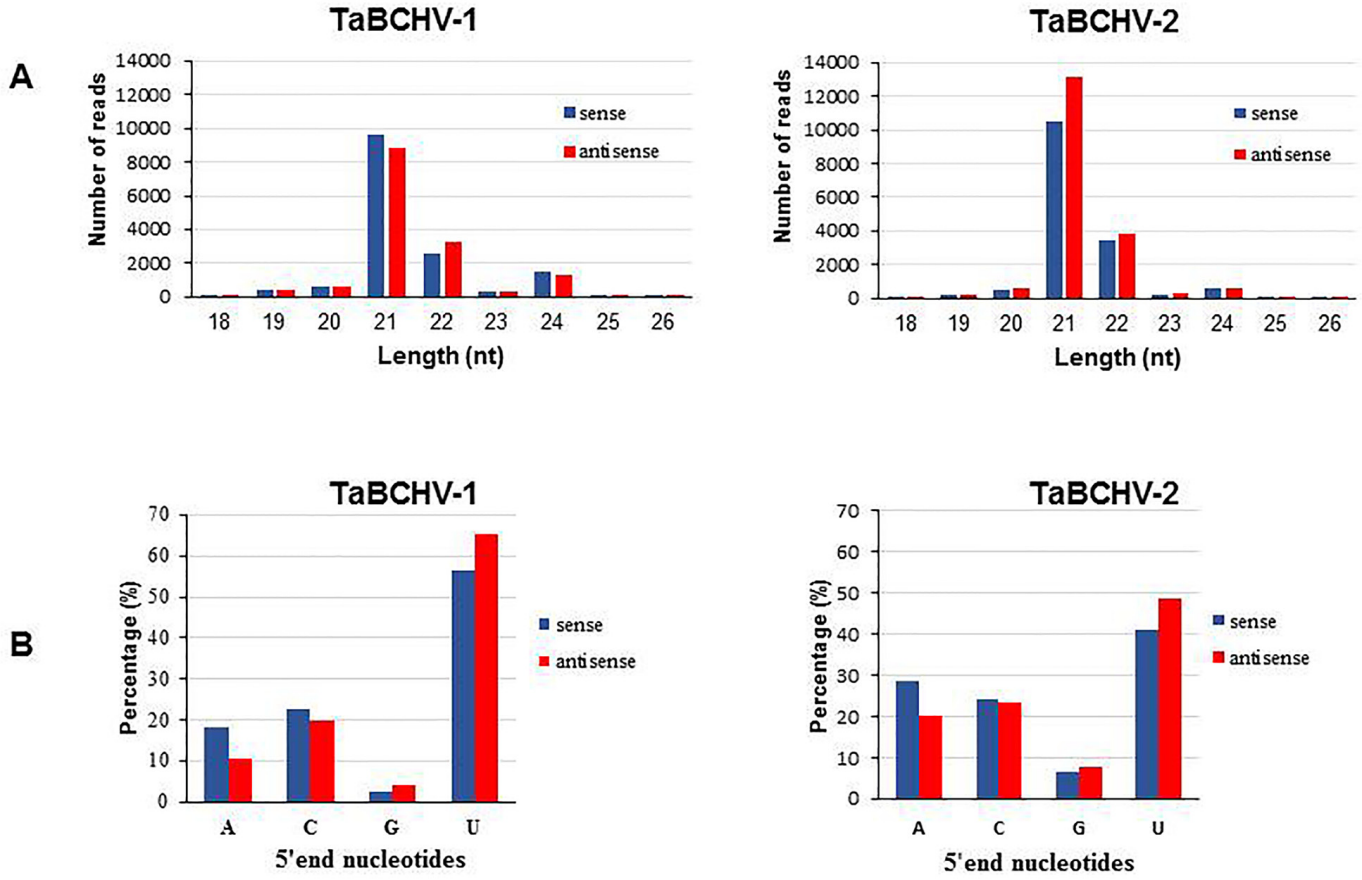
Size distribution of vsRNAs derived from TaBCHV-1 and TaBCHV-2 (A) and the relative frequency of 5' terminal nucleotide of 21- and 22-nt vsRNA (B). Blue and red bars indicate sense and antisense vsRNAs respectively.

In total, 23,708 and 52,121 vsRNAs of 18‒26 nt, accounting for 0.25% and 0.53% of the total reads, matched the genome sequences of TaBCHV-1 and TaBCHV-2, respectively. The size classes of vsRNAs from sense and antisense strands of both isolates were mostly within the range of 21–24 nt, with 21-nt vsRNAs being a predominant class, followed by 22-nt vsRNAs (Fig 4A). Analysis of the 5’-terminal nucleotide in 21- and 22-nt sRNAs derived from both TaBCHV-1 and TaBCHV-2 revealed that U was the most prevalent and G was the least abundant regardless the polarity of their genome strains (Fig 4B).

**Fig 5:**
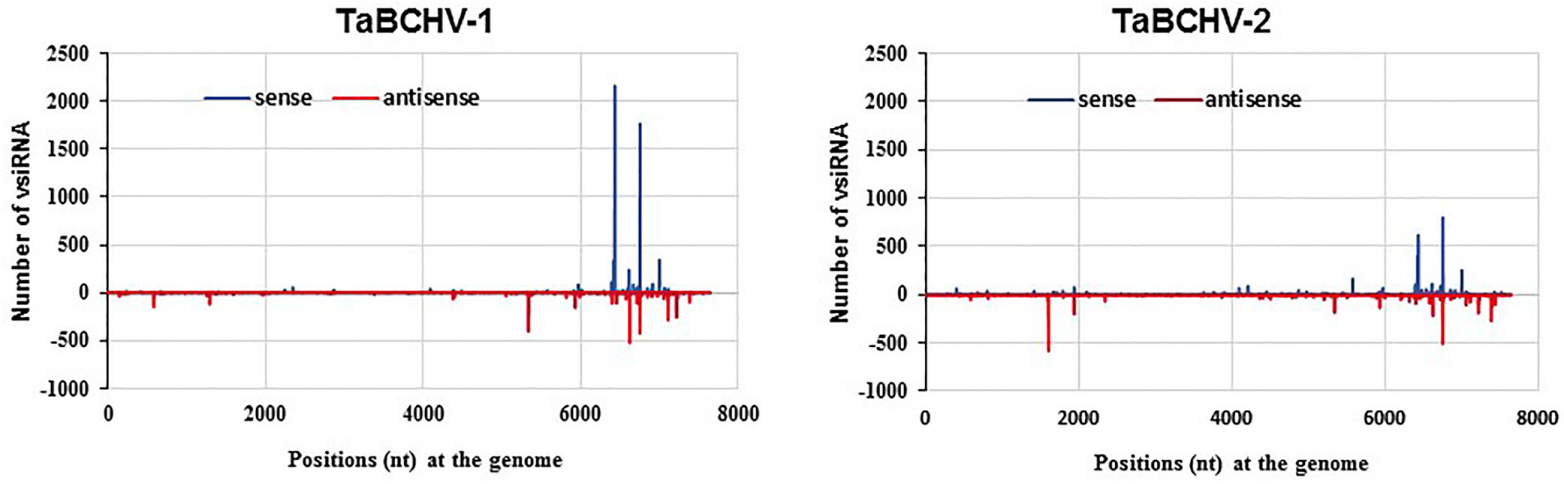
The distribution of 21- and 22-nt vsRNAs on the genomes of TaBCHV-1 and TaBCHV-2. The bars above the axis represent sense reads; those below represent antisense reads.

**Fig 6:**
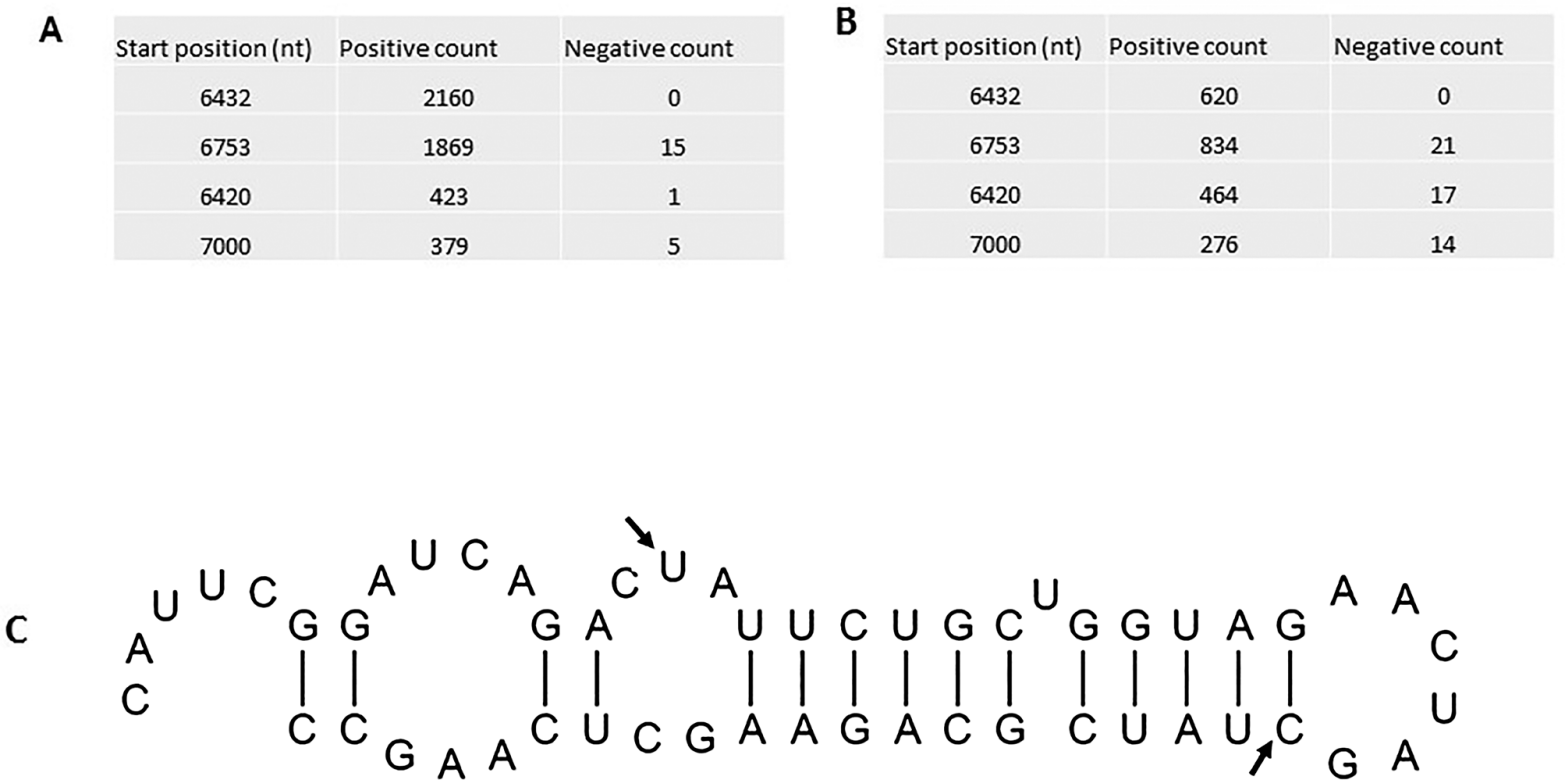
The silencing hot spots of TaBCHV-1(A) and TaBCHV-2(B) genomes and the predicated secondary structure around the vsRNA at 6432 nt (C). The start and stop positions of the vsRNA_6432_ are marked by bold arrows.

There was no significant difference of the amount of the 21- and 22-nt vsRNAs mapped to the sense and antisense strands of the viral genome, and the vsRNAs from both senses were discontinuous and unevenly distributed along the viral genome (Fig 5). Meanwhile, one hotspot region located within ORF3 was identified. In the region, two vsRNAs started at 6432 nt and 6753 nt of TaBCHV-1 and TaBCHV-2 genome were highly repeated (Fig 6A and 6B). The secondary structure analysis of a 50-nt sequence around the hotspot by using the RNAfold program (http://rna.tbi.uninie.ac.at/cgi-bin/RNAfold.cgi) revealed that the sequence around the vsRNA_6432_ could form a highly structured stem-loop (Fig 6C), indicating that the secondary structure might contribute to the production of the vsRNA [40].

## Discussion

Deep sequencing of sRNAs is a powerful tool to identify the consensus and specific sRNAs. In the present study, a novel badnavirus, TaBCHV was identified by sRNA sequencing of viral infected taro plants. Based on the sequences of contigs assembled from sRNAs of two taro samples and in combination with sequence specific PCR amplification, the genome sequences of two TaBCHV isolates were determined for the first time. The virus showed a genomic structure that was similar to other badnaviruses by having three tandemly arranged ORFs on the sense strand [41] and a large polycistronic transcript functioning as MP, CP, AP, RT, and RNaseH, which are critical to the dsDNA viral lifecycle.

Genomic structure and sequence analyses revealed that TaBCHV possesses unique characteristics. On the sense strand of its genome were six ORFs, of which two additional ORFs (ORF5 and ORF6) were absent in the genome of TaBV, which is a known badnavirus infecting taro plants [3]. The positions of ORF5 and ORF6 were also different from that of some other badnaviruses. The TATA box (TATAAA) located at position 7,503‒7,508 nt, and a downstream poly adenylation signal (AAAATAA) at position 7,624‒7,630 nt, which are consider to be essential for the production of a terminally redundant full-length transcript [42], were detected in the TaBCHV genome, but the poly adenylation signal was not detected in the TaBV genome [3]. Then, the function of poly adenylation signal should be further addressed. Furthermore, the sequences of all predicated coding regions were highly different from those of other badnaviruses. Our previous results showed that the 576-bp fragments covering a partial RT/RNase H region from Chinese taro badnavirus isolates shared 78.8% ‒ 99.5% similarity at the nt level and 81.3% ‒ 99.5% at aa level [7]. Taken together, these results support that TaBCHV is a new species of the genus *Badnavirus*, based on the standards for differentiating badnaviruses that was established by the ICTV [43].

To date, several badnaviruses have been reported as targets of the RNA silencing machinery of various host plants. Analysis of sRNAs derived from the genomes of two TaBCHV isolates revealed that 21-nt vsRNAs were predominant, followed by 22-nt vsRNAs, suggesting that the taro homologue of DCL4 and DCL2 might be the predominant Dicer ribonucleases involved in vsRNA biogenesis [44]. The results were similar to those obtained from PYMAV and *Banana streak gold finger virus* (BSGFV), *Banana streak Imove virus* (BSIMV), *Banana streak Veitnam virus* (BSVNV), *Banana streak Cavendish virus* (BSCAV), and, in which both 21-nt and 22-nt sRNAs were the most prevalent [22, 45], but different from that of SPBV-A, SPBV-B and RYNV [20, 23]. The bias observed at the 5' termini of TaBCHV sRNAs was similar to that observed in the BSV species [45]. These results suggest that 21-nt vsRNAs might be potentially loaded into diverse AGO containing complexes, with most of the vsRNAs preferentially recruited into AGO1 and AGO4, which showed a preference for the nucleotide “U” [46].

The present study observed an uneven distribution of 21-nt sRNAs in the TaBCHV genome in both polarities, with major hotspots in the ORF5 region that were different from the BSV hotspots that were concentrated in ORF1 and ORF2. Also, hotspots facilitated in the generation of stable secondary structures that might serve as an effective means for evading RNA silencing [47]. However, the significance of hotspots and the associated hairpin structures in affecting virus infectivity or in interfering with host gene expression remains elusive and thus requires further investigation.

## Supporting Information

S1 Table Primers used for full genome amplification and detection of TaBCHV. (DOCX)

S2 Table Members of the genus *Badnavirus* and *Tungrovirus* used for phylogenetic analysis. (DOCX)

S3 Table Coding capacity of TaBCHV open reading frames. (DOCX)
