# Supplementary material for: Characterization by Small RNA Sequencing of Taro Bacilliform CH Virus (TaBCHV), a Novel Badnavirus
Source: PLoS One. 2015 Jul 24;10(7):e0134147. doi: 10.1371/journal.pone.0134147 (PMC4514669; doi:10.1371/journal.pone.0134147)
Supplement: S1 Table — (DOCX) [file pone.0134147.s001.docx]

**S1 Table. Primers used for full genome amplification and detection of both isolates of TaBCHV**

| **Primer name** | **Primer sequences （5**'–**3**'**）** | **Length (bp)** |
| --- | --- | --- |
| CF1 | ACAAGCGATCCAGATCTCC | 396 |
| CR1 | CTCTCTAGGCTTTTGTTCGGAT |  |
| CF2 | GAAGCTATCCGAACAAAAGCCTA | 755 |
| CR2 | CAAGCTTCATATCCTCGGGTG |  |
| CF3 | ATTTGCGAACGAAGTCCAGA | 1200 |
| CR3 | CATGTTTTGGATGCAAGCCT |  |
| CF4 | CCCCTACCAAGGATCCACA | 601 |
| CR4 | TCTTGAACGGAGACTATATCACA |  |
| CF5 | AAATCTGGACCTACCCGAGGA | 935 |
| CR5 | GAATTCGAAAGGCATTATCACC |  |
| CF6 | TCATTGGTGATAATGCCTT | 612 |
| CR6 | AGAGCGTATGGTCTTCAATT |  |
| CF7 | AAAATATACTCCAAATTCGACCT | 1009 |
| CR7 | ATAAGTAGCTCCTTCTTGTCA |  |
| CF7 | TTTCAGCACATTGATGGCAAA | 2001 |
| CR8 | GATCGCTTGTTCTTCTACCA |  |
| TF1 | TATGAAGAACCATTTGCGTGA | 923 |
| TR1 | CTGTTAGCCTTACAAGCAAC |  |
| TF2 | AGGAAATAGCAGCACAACCAA | 1118 |
| TR2 | TTGTAGAAATCCCCAATAGTCA |  |
| TF3 | CTTCTCTGCACCGACAACCAA | 1364 |
| TR3 | CACCATTCGGAGTAGTCCT |  |
| TF4 | CACTACGAGTAATCCGACCAG | 1157 |
| TR4 | CCCTCCGATACAGTATGTCT |  |
| TF5 | GTTATGTCTGTCTCCGAAC | 1261 |
| TR5 | TGTGTCGCTTATTAGATGCTT |  |
| TF6 | CCAATAAAATTACGTGCCAAC | 1398 |
| TR6 | GATCCGAATGAAAATCTGGTT |  |
| TF7 | AAAGACAACGCATTAGCTG | 1398 |
| TR7 | TTATTCATCCAGTCACGCAAA |  |
