# Supplementary material for: Characterization by Small RNA Sequencing of Taro Bacilliform CH Virus (TaBCHV), a Novel Badnavirus
Source: PLoS One. 2015 Jul 24;10(7):e0134147. doi: 10.1371/journal.pone.0134147 (PMC4514669; doi:10.1371/journal.pone.0134147)
Supplement: S2 Table — (DOCX) [file pone.0134147.s002.docx]

**S2 Table. Members of the genus *Badnavirus* and *Tungrovirus* used for phylogenetic analysis**

| **Species names (Acronym)** | **Genus** | **Accession No.** |
| --- | --- | --- |
| *Banana streak virus* (BSV) | *Badnavirus* | NC_008018 |
| *Banana streak IM virus* (BSIMV) | *Badnavirus* | NC_015507 |
| *Cacao swollen shoot virus* (CSSV) | *Badnavirus* | NC_001574 |
| *Citrus yellow mosaic virus* (CYMV) | *Badnavirus* | NC_003382 |
| *Commelina yellow mottle virus* (CoYMV) | *Badnavirus* | NC_001343 |
| *Cycad leaf necrosis virus* (CyLNV) | *Badnavirus* | NC_011097 |
| *Dioscorea sansibarensis bacilliform virus* (DsBV) | *Badnavirus* | NC_009010 |
| *Dracaena mottle virus* (DrMV) | *Badnavirus* | NC_008034 |
| *Fig badnavirus*  (FBV) | *Badnavirus* | NC_017830 |
| *Gooseberry vein banding virus* (GVBV) | *Badnavirus* | NC_018105 |
| *Grapevine vein-clearing virus* (GVCV) | *Badnavirus* | NC_015784 |
| *Hibiscus bacilliform virus* (HBV) | *Badnavirus* | NC_023485 |
| *Kalanchoe top-spotting virus* (KTSV) | *Badnavirus* | NC_004540 |
| *Pagoda yellow mosaic associated virus* (PYMAV) | *Badnavirus* | NC-024301 |
| *Pelargonium vein banding virus* (PVBV) | *Badnavirus* | NC_013262 |
| *Piper yellow mottle virus* (PYMoV) | *Badnavirus* | NC_022365 |
| *Pineapple bacilliform comosus virus* (PBCoV) | *Badnavirus* | NC_014648 |
| *Sugarcane bacilliform IM virus* (SBIMV) | *Badnavirus* | NC_003031 |
| *Rubus yellow net virus* (RYNV) | *Badnavirus* | KF_241951 |
| *Taro bacilliform virus* (TaBV) | *Badnavirus* | NC_004450 |
| *Rice tungro bacilliform virus* (RTBV) | *Tungrovirus* | NC_001914 |
