# Supplementary material for: Characterization by Small RNA Sequencing of Taro Bacilliform CH Virus (TaBCHV), a Novel Badnavirus
Source: PLoS One. 2015 Jul 24;10(7):e0134147. doi: 10.1371/journal.pone.0134147 (PMC4514669; doi:10.1371/journal.pone.0134147)
Supplement: S3 Table — (DOCX) [file pone.0134147.s003.docx]

**S3 Table. Coding capacity of TaBCHV open reading frames**

| **ORF** | **First nucleotide** | **Last nucleotide** | **No. amino acids** | **Molecular mass (kDa)** |
| --- | --- | --- | --- | --- |
| 1 | 384 | 821 | 145 | 16.8 |
| 2 | 818 | 1198 | 126 | 14.1 |
| 3 | 1192 | 6609 | 1805 | 206.4 |
| 4 | 2096 | 2455 | 119 | 13.2 |
| 5 | 6530 | 6838 | 102 | 11.9 |
| 6 | 6720 | 7043 | 107 | 12.4 |
